# Aquaporin-4 expression in the human choroid plexus

**DOI:** 10.1007/s00018-022-04136-1

**Published:** 2022-01-24

**Authors:** Felix Deffner, Corinna Gleiser, Ulrich Mattheus, Andreas Wagner, Peter H. Neckel, Petra Fallier-Becker, Bernhard Hirt, Andreas F. Mack

**Affiliations:** 1 Institute of Clinical Anatomy and Cell Analysis, University of Tübingen, Österbergstr. 3, 72074 Tübingen, Germany; 2 Institute of Pathology and Neuropathology, University of Tübingen, Tübingen, Germany

**Keywords:** Ependyma, Cerebrospinal fluid, Aquaporin, Choroid plexus, Astroglia

## Abstract

The choroid plexus (CP) consists of specialized ependymal cells and underlying blood vessels and stroma producing the bulk of the cerebrospinal fluid (CSF). CP epithelial cells are considered the site of the internal blood-cerebrospinal fluid barrier, show epithelial characteristics (basal lamina, tight junctions), and express aquaporin-1 (AQP1) apically. In this study, we analyzed the expression of aquaporins in the human CP using immunofluorescence and qPCR. As previously reported, AQP1 was expressed apically in CP epithelial cells. Surprisingly, and previously unknown, many cells in the CP epithelium were also positive for aquaporin-4 (AQP4), normally restricted to ventricle-lining ependymal cells and astrocytes in the brain. Expression of AQP1 and AQP4 was found in the CP of all eight body donors investigated (3 males, 5 females; age 74–91). These results were confirmed by qPCR, and by electron microscopy detecting orthogonal arrays of particles. To find out whether AQP4 expression correlated with the expression pattern of relevant transport-related proteins we also investigated expression of NKCC1, and Na/K-ATPase. Immunostaining with NKCC1 was similar to AQP1 and revealed no particular pattern related to AQP4. Co-staining of AQP4 and Na/K-ATPase indicated a trend for an inverse correlation of their expression. We hypothesized that AQP4 expression in the CP was caused by age-related changes. To address this, we investigated mouse brains from young (2 months), adult (12 months) and old (30 months) mice. We found a significant increase of AQP4 on the mRNA level in old mice compared to young and adult animals. Taken together, we provide evidence for AQP4 expression in the CP of the aging brain which likely contributes to the water flow through the CP epithelium and CSF production. In two alternative hypotheses, we discuss this as a beneficial compensatory, or a detrimental mechanism influencing the previously observed CSF changes during aging.

**Supplementary Information:**

The online version contains supplementary material available at 10.1007/s00018-022-04136-1.

## Introduction

The internal environment in the central nervous system (CNS) is separated from blood and surrounding tissues by several barrier-forming structures. Besides the blood–brain barrier, there is an outer and inner blood-cerebrospinal fluid barrier (BCSFB), the outer BCSFB formed by the arachnoid cells, the inner by the choroid plexus epithelial cells (CPCs). The choroid plexus (CP) itself is a structure found in all brain ventricles (I-IV) [1] and thought to be the main production site of the cerebrospinal fluid (CSF). The CP consists of blood vessels, the overlaying epithelial cells, and varying amounts of stromal cells and matrix in between. CPCs express ion channels, transport proteins, and tight junction proteins which form the actual BCSBFB, reviewed in [2, 3].

Besides its main role in CSF production, the CP has been attributed with other functions such as water homeostasis, endocrine regulation, immune surveillance [4–6], and secretion of stimulating stem cells factors [7]. In addition, the CP might be used as an entry site for parasites [8] or viruses including SARS-CoV2 into the brain [9].

The CPCs can be considered as specialized ependymal cells which otherwise cover the walls of the ventricles, since both originate from neuroepithelium [10] and form a continuous sheet of cells [11]. However, 'regular' ependymal cells differ from CPCs structurally and molecularly: ependymal cells do not form tight junctions in mammals, are derived from radial glia, and mostly bear kinocilia whereas CPCs mostly have apically located microvilli and form a true epithelium attached to a basal lamina. Moreover, many transport and channel proteins on CPCs are involved in the CSF production.

In this study, we first focused on the distribution of water channel proteins in ependymal cells and CPCs of the human choroid plexus. It has been well established that CPCs express aquaporin-1 (AQP1) apically. The first identified water channel was AQP1, at the time called CHIP, and was localized in the brain on choroid plexus cells [12]. Subsequently, another aquaporin abundantly expressed in the brain was discovered, designated aquaporin-4 (AQP4) [13] which was then localized to astrocytic endfeet and ependymal cells [14]. This localization coincided with the square arrays or orthogonal arrays of particles (OAPs) [15] detected by freeze-fracture electron microscopy prior to the discovery of AQP4 (reviewed then in [16]). Since then, many aspects on the functions of aquaporins in the brain have been studied (see reviews by [17–20]) including implications in brain diseases [21]. More recently, the role of aquaporins in the brain have been implicated with the homeostasis of cerebrospinal fluid (CSF) [22], and the glymphatic waste removal system, also with respect to aging [23].

Whereas ependymal cells show a basolateral localization of AQP4, the expression of AQP1 on CPCs is mostly apical. Given the continuity of the ependymal ventricular lining and CPCs, the initial goal of the present study was to establish where in the human ependyma-CP transition the AQP expression would switch from AQP4 to AQP1. Unexpectedly, we discovered that in the brains of body donors, there was also expression of AQP4 in CPCs. We compared this expression pattern to the aquaporin distribution and expression in the murine choroid plexus and ependyma of different age groups.

## Materials and methods

### Post-mortem specimens

Human CP were taken from eight individuals who voluntarily donated their bodies to the Institute of Clinical Anatomical and Cell Analysis, Tübingen. They gave their informed consent in agreement with the declaration of Helsinki to use the cadaver for research purposes. This procedure was approved by the Ethics Committee of the Medical Department of the University of Tübingen under the project number 237/2007BO1. Details on the female and male body donors aged 74 and 94 years are provided in supplementary table 1. CP samples were collected and processed within 8 to 19 h post-mortem. Parenchyma from the striatum was used as reference tissue for histology and RNA isolation.

### Animals

For this study we used the CP from C57BL/6 mice bred in the facility of our institute. All procedures were performed according to University of Tübingen and governmental guidelines, and were approved by local authorities (Regierungspräsidium Tübingen). For PCR analysis, we used 15 mice from both sexes and three age cohorts: young (2–6 months old), adult (12 months old) and old (more than 30 months old), five animals each. Immunohistochemistry was performed on tissues from ten animals. For the removal of the CP and parenchyma from the striatum, mice were anaesthetized with CO_2_ and decapitated.

### RNA isolation

For qPCR analysis, the CP was removed immediately and placed in ice-cold, RNAse-free phosphate buffered saline (PBS, Sigma Aldrich, St. Louis, MO, USA). Care was taken to obtain solely tissue from the CP. Unfortunately, a pure CP epithelial preparation cannot be performed in such a way that no surrounding tissue is attached, especially for the mouse CP. For this reason, parenchyma from the striatum was used as control tissue for human and murine brains. For each prepared mouse, CPs from the left and right lateral ventricles were combined. In addition, the CP from the fourth ventricle was collected as a single sample, so that two CP samples were obtained from each mouse. The reference striatal tissue was always taken from the corresponding brain.

### Immunohistochemistry

After removal, the excised CP was fixed in 4% paraformaldehyde overnight, afterwards rinsed with PBS, and placed into 30% (w/v) sucrose for another 24 h for cryoprotection. The fixed samples were frozen in isopentane-nitrogen cooled TissueTek^®^ (Sakura, Staufen, Germany), stored at -80 °C before cryosectioned at 18 µm.

Sections were re-hydrated and washed in PBS for 10 min, followed by incubation in blocking solution containing PBS, 4% (v/v) goat serum (Biochrom, Berlin, Germany), 0.1% (v/v) bovine serum albumin (Roth, Karlsruhe, Germany), and 0.1% (v/v) Triton^®^ X-100 (Roth, Karlsruhe, Germany) for 90 min. at room temperature. Next, the sections were incubated with primary antibodies (Table 1) diluted in the preincubation solution overnight at 4 °C in a humidified chamber. After washing with PBS three times for 10 min, the secondary antibodies (Table 1) were applied for 90 min at room temperature. Afterwards, sections were stained with the nuclear stains DRAQ5 (1:1000; Thermo Fisher, Waltham, MA, USA) or DAPI (1:1000) and washed with PBS three times for 10 min before mounting with Mowiol 4–88 (Roth).

**Table 1:** Primary and secondary antibodies used in this study

Antibody	Host	Dilution	Source
Primary AB			
AQP1	Mouse	1:100	Santa Cruz Biotechnology, Dallas, USA
AQP1	Rabbit	1:100	Thermo Fisher Scientific, Walmart, USA
AQP4	Rabbit	1:100	Santa Cruz Biotechnology, Dallas, USA
Na/K-ATPase	Mouse	1:100	Hybridoma Bank, Iowa, USA
NKCC1	Mouse	1:100	Abcam, Cambridge, England
Laminin	Rabbit	1:50	Abcam, Cambridge, England
Secondary AB			
Anti-mouse Alexa 488	Goat	1:400	Invitrogen, CA, USA
Anti-mouse Alexa 546	Goat	1:400	Invitrogen, CA, USA
Anti-rabbit Alexa 488	Goat	1:400	Invitrogen, CA, USA
Anti-rabbit Alexa 546	Goat	1:400	Invitrogen, CA, USA

### Light microscopy

The cryostat sections were analyzed on a Zeiss LSM510 Meta confocal microscope (Zeiss, Oberkochen, Germany) equipped with an argon laser excitation wavelength at 488 nm and two helium–neon lasers with wavelengths for excitation at 543 nm and 633 nm, respectively and appropriated filter set. Alternatively, images were taken on an Axio Imager Z1 fluorescence microscope (Zeiss) with an Apotome module. The systems’ software Black and Blue ZEN were used for image acquisition, and image plates were assembled and processed with Adobe Photoshop CS2 (San José, CA, USA).

### Electron microscopy

CP tissue was fixed in 2.5% glutaraldehyde buffered in 0.1 M cacodylate (pH 7.4) for 2 h. For ultrathin sections, samples were post-fixed in 1% osmium tetroxide in PBS (pH 7.4) for 1 h and subsequently dehydrated in a graded ethanol series and acetone, and embedded in epoxy resin (Sigma Aldrich, Darmstadt, Germany).

For freeze-fracture sample preparation, fixed tissues were cryoprotected in 30% glycerol and snap-frozen in nitrogen slush (-210 °C). Subsequently, they were fractured in a freeze fracture apparatus (BAF400D; Balzers, Liechtenstein) at 5 × 10^–6^ mbar and -150 °C. The fracture faces were contrasted with platinum/carbon (3 nm, 45°) and stabilized with carbon (30 nm, 90°) for stabilization of the replica. Remaining cell material was removed with 12% sodium hypochlorite, and the rinsed replicas were collected on Pioloform-coated copper grids.

Ultrathin sections and freeze-fracture replicas were analyzed, and images recorded on a Zeiss EM10 or a LEO 912AB transmission electron microscope (both Zeiss, Oberkochen, Germany).

### RT and qPCR analysis

Preparation material was placed in a Precellys^®^ Lysing Kit (VWR Life Science Competence Center, Erlangen, Germany) filled with 900 µl QIAzol^®^ Lysis Reagent (Qiagen, Hilden, Germany) immediately after collection and placed on ice. After thawed on ice, the samples were homogenized for 10 s at 3000 rpm in a Minilys system (Bertin Instruments, Montigny-le-Bretonneux, France). The homogenized samples were stored at −80 °C until further processing.

The collected tissues were incubated for 5 min at room temperature. After addition of 100 µl gDNA eliminator (Qiagen), the homogenate was transferred to a MaXtract High Density Tube (Qiagen) and 200 µl chloroform was added. Centrifugation was performed at 12,000 rpm and 4 °C for 5 min. The upper aqueous phase containing the nucleic acids was pipetted into a new Eppendorf reaction tube (2 ml Safe-Lock). The RNA was automatically isolated in a QIAcube^®^ (Qiagen) using the RNeasy^®^ Plus Universal Mini Kit (Qiagen) and the corresponding QIAcube^®^ (Qiagen) protocol.

The QIAxcel Advanced System (Qiagen) was used to determine both RNA integrity and RNA concentration. Only RIS values (RNA integrity score) of at least 6.0 were used for murine samples, and RIS values of at least 5.8 were used for human samples.

The Reverse Transcription was performed with the QuantiTect Reverse Transcriptase Kit (Qiagen) according to the manufacturer's instructions. For negative controls, the Reverse Transcriptase was replaced with nuclease-free water. The total cDNA concentration of each sample was measured using the Qubit™ ssDNA Assay Kit on a Qubit 2.0 Fluorometer (Thermo Fisher Scientific, Waltham, MA, USA).

The primers/probes used to quantify mRNA expressions of aquaporin genes were acquired from TaqMan^®^GenExpression assays (Thermo Fisher) as summarized in supplementary table 2.

cDNA with a concentration of 5 ng/µl was used for analysis. Measurements were conducted in triplicates, and a no-template blank served as the negative control (duplicates). Parenchyma from the striatum was used as reference tissue. qPCR was performed on Applied Biosystems Step One™ (Applied Biosystems—Thermo Fisher) for 40 cycles: 15 s denaturation at 95 °C, followed by 1 min annealing at 60 °C. The data were collected with the StepOne™ Software v2.3 and Ct values were exported to Microsoft Excel.

### qPCR data evaluation and statistical analysis

Analysis of relative mRNA expression was performed using qbase + software (Biogazelle, Zwijnaarde, Belgium), with relative abundance (RQ values) calculated using a series of normalization methods based on the classical delta-delta Ct method and MIQE—compliant procedures [24]. The RT-qPCR cycle threshold (Ct) values were the input data in the qbase + program. Results were calculated for 100% PCR efficiency and ‘unpaired’ experimental design.

Statistical analysis for mouse AQP4 expression was performed with GraphPad Prism 6.07 (GraphPad Software, San Diego, USA) by processing the qbase + RQ values using an unpaired t test. P values < 0.05 were considered statistically significant.

After the amplification, PCR products were analyzed by high-resolution capillary electrophoresis with the Qiaxcel DNA High Resolution Kit, QX Alignment Marker 15 bp/600 bp and the QX DNA Size Marker 25–500 bp at a concentration of 30 ng/µl was used (Qiagen). The separation was performed using the OM800 method of the Qiaxcel System with the following parameters: 4 kV and 5 s for alignment marker injection, 5 kV and 10 s for the sample injection and 3 kV for 800 s for separation. The results were displayed as gel images as obtained from QIAxcel system software.

## Results

### Localization of aquaporins in the choroid plexus

**Fig. 1:**
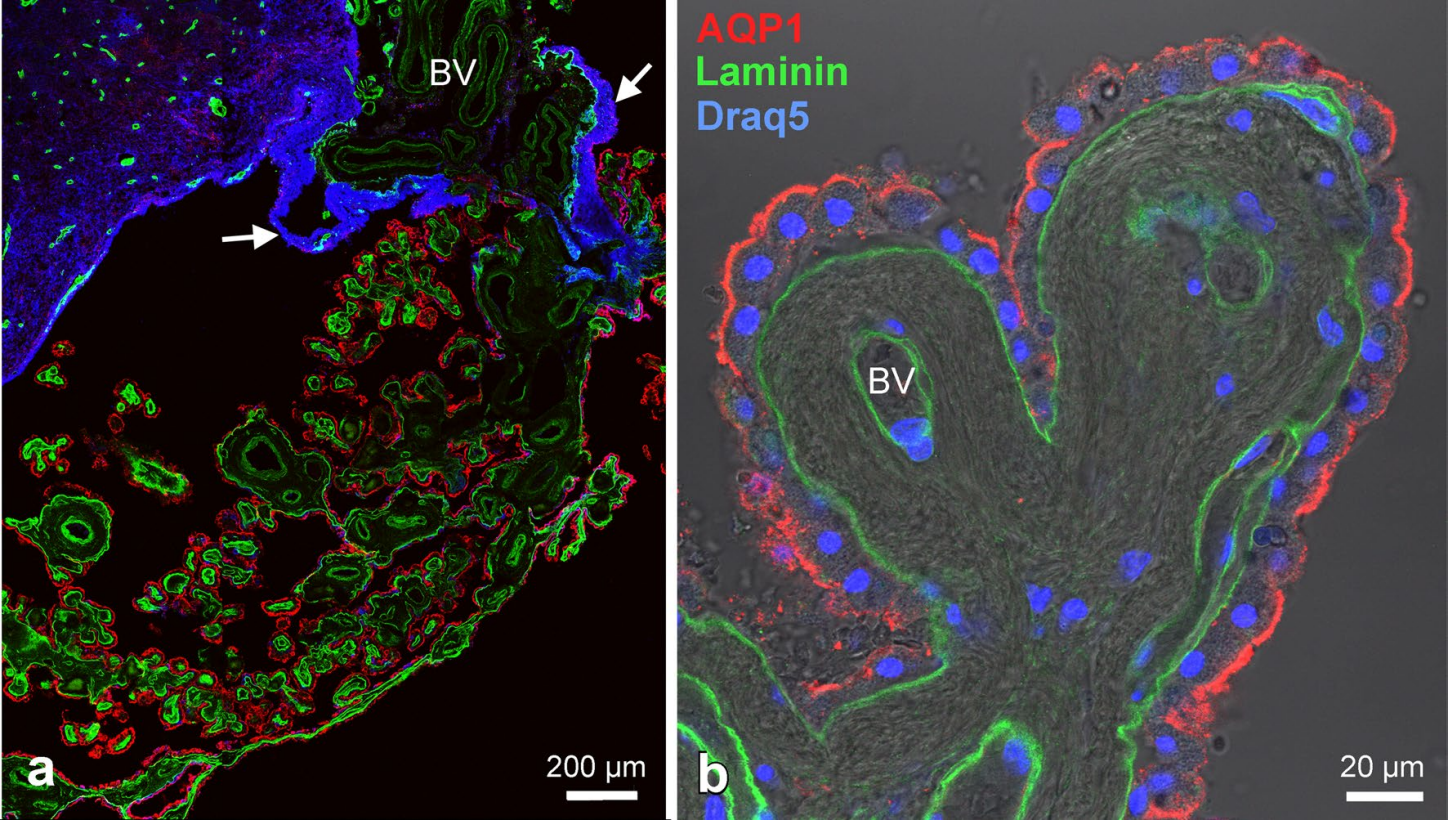
Human choroid plexus (CP) and ependyma in the lateral ventricle immunostained for AQP1 (red) and laminin (green), cell nuclei labeled with Draq5 (blue). **a** Overview of the transition zone between the CP and the ependymal lining of brain parenchyma. AQP1 is restricted to the CP epithelium and is not present in ependymal cells. Laminin, as part of the basal lamina (BL), is found in the CP in the BL of the epithelium, and in the BL of the blood vessels (BV) in the stroma of the CP and brain parenchyma. Note that the ependymal lining does not have a basal lamina (except where BV are present). BV supplying the CP are surrounded by astroglia and ependyma in the transition zone (arrows). **b** Close-up view of a CP villus. AQP1 is expressed continuously mostly on the apical side of the CP epithelial cells. The laminin staining indicates a thick stromal layer of connective tissue between the two BLs highlighting a considerable diffusions distance

In this study, we focused on the distribution pattern of water channels and other transport proteins in the human CP. First, to investigate the transition area between the CP epithelium and ependyma, we stained for the water channel aquaporin-1 (AQP1) and the extracellular protein laminin, an essential component of the basal lamina (Fig. 1). As expected, AQP1 was strongly expressed by plexus epithelium cells in the apical membrane domain, in some cells there was also a weak expression basolaterally. Occasionally, a reduction or even complete absence of AQP1 was observed. Laminin showed a continuous expression delineating the basal lamina of the CP epithelium (Fig. 1a). The CP epithelium covers underlying blood vessels embedded in connective tissue, collectively called the *Tela choroidea*. The endothelial layer of capillaries and the *Tunica intima* of larger blood vessels is delimited by a basal lamina as well. Thus, in many CP villi, two basal laminae (epithelial and endothelial) were observed separated by a thick layer of connective tissue (Fig. 1b). At the entrance of blood vessels into the choroid plexus, the endothelial basal lamina was continuous whereas the epithelial basal lamina appeared in the transition zone where ependymal cells connect with the CP epithelium. Along a similar gradient, the AQP1 immunoreactivity in the transition zone covering epithelium became patchy and was entirely lacking in the ependymal lining. Thus, we confirmed previous findings of the absence of AQP1 and a basal lamina in the ependyma, and their presence in CP epithelium.

**Fig. 2:**
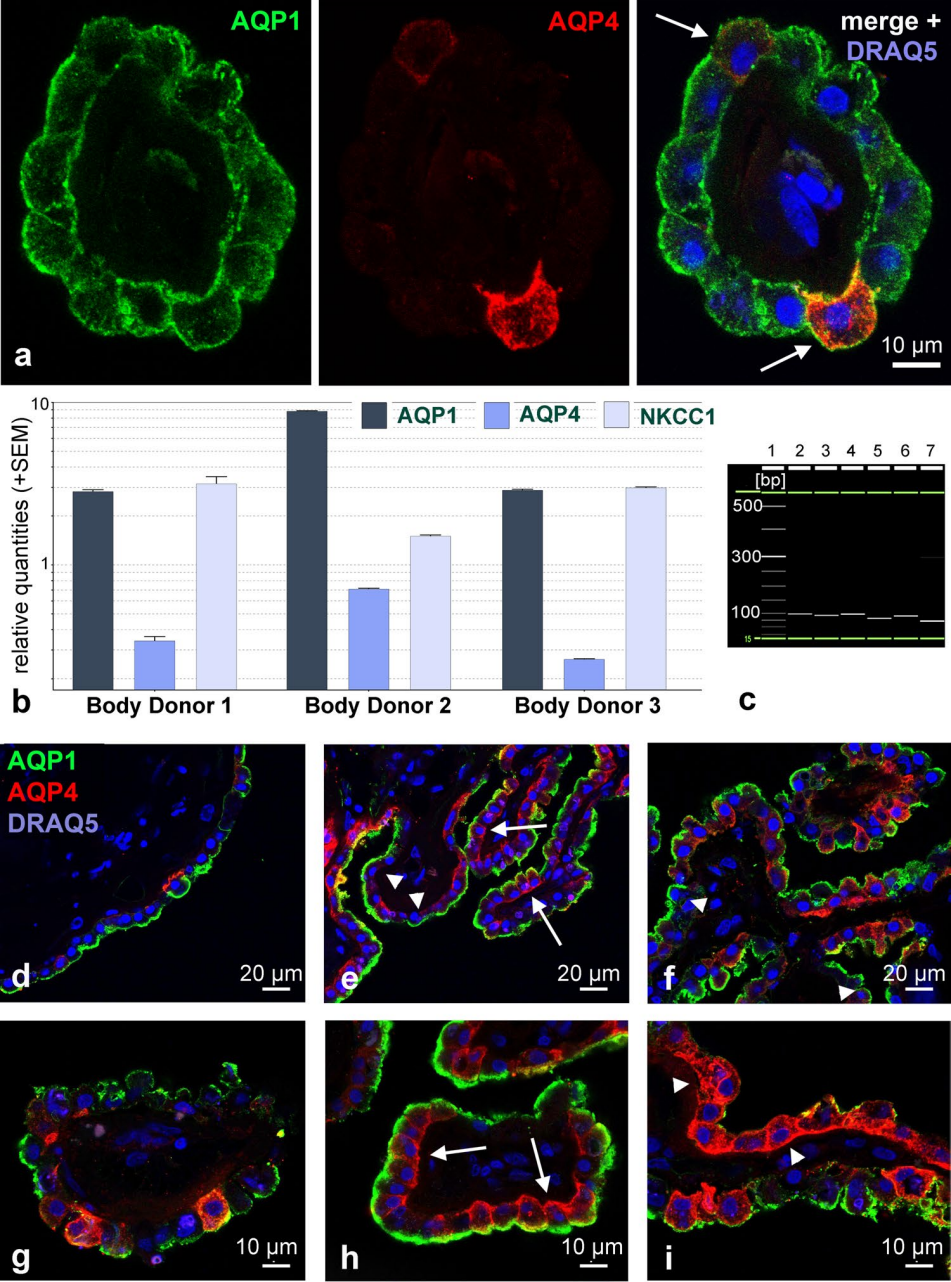
Expression of AQP1 and AQP4 in the human choroid plexus. **a** A CP villus in the lateral ventricle stained for AQP1 (green) and AQP4 (red). The arrows indicate CPCs expressing AQP4 mostly basolaterally whereas AQP1 is expressed mostly apically. **b** Gene expression analysis for AQP1, AQP4, and NKCC1 using TaqMan^®^ assays of three human body donors, HPRT, TBP and UBC served as reference genes. Besides the expected AQP1 mRNA and NKCC1 mRNA, AQP4 mRNA was present in the samples of all three body donors. **c** Exemplary gel image of RT-PCR products obtained from the QIAxel system software showing the expected product size for AQP1 (96 bp), AQP4 (92 bp), HPRT (82 bp), TBP (91 bp), UBC (71 bp), NKCC1 (97 bp). Lane 1-size marker, 2-AQP1, 3-AQP4, 4-NKCC1, 5-HPRT, 6-TBP, 7-UBC. **d–i** Distribution pattern of AQP1 and AQP4 on cryostat sections of the human CP in the lateral ventricle from different body donors. There were areas (**d**, **e**) with single AQP4-positive cells, and areas with clusters of cells expressing AQP4 (**h**, **i)**, over 50% of all epithelial cells in this region). When investigating cellular localization, we found cells expressing both AQP1 and AQP4 (**e**, **h**; arrows), as well as cells expressing only one of the two water channels (**e**, **f**, **i**; arrow heads)

Since it is known that the ependyma expresses the water channel aquaporin-4 (AQP4) in the basolateral membrane domain, we stained the human CP and ependyma for both aquaporins, AQP1 and AQP4. As expected, AQP4 was clearly located on ependymal cells. Surprisingly however, we found AQP4 positive cells in the CP of all human body donors (Fig. 2). This AQP4 immunofluorescence was in most cells located in the basolateral membrane domain. Additionally, in few CP epithelial cells there was a remarkable immunoreactivity in the cytoplasm as well as in the membrane (Fig. 2a).

### Aquaporin mRNA detection in the choroid plexus

To gather further evidence for the expression of AQP4 in the human CP, we performed a gene expression analysis using TaqMan^®^ assays on tissues from three human body donors (Fig. 2b). In all three human CP samples, we found AQP4 mRNA to be expressed even though in a lower amount than AQP1 mRNA. In contrast, the striatal control tissue showed high levels of AQP4 mRNA expression and very small relative amounts of AQP1 mRNA (SM Fig. 1). We confirmed the specificity of amplified PCR products by QIAxcel high-resolution capillary electrophoresis showing the expected product size for AQP1, AQP4, NKCC1, and the three reference genes (Fig. 2c). This clearly demonstrates the expression of AQP4 at the mRNA level in the CP.

### Distribution of aquaporin-4 expression

Immunostaining and evaluating sections of the CP from eight body donors for AQP1 and AQP4, we could not detect a particular pattern in any of the samples with the following observations: We found cells that expressed AQP4, and these cells appeared as individual cells or in groups. These AQP4 positive cells were also immunoreactive for AQP1, i.e. double labeled, or they were lacking AQP1 immunoreactivity. Examples of this heterogenous distribution are shown in Fig. 2d–i.

We attempted to correlate the expression of AQP4 with the occurrence of psammoma bodies or amounts of increased connective tissue in the stroma under the CP epithelial cells but found no clear evidence for such a relationship.

### Ultrastructural detection of AQP4

**Fig. 3:**
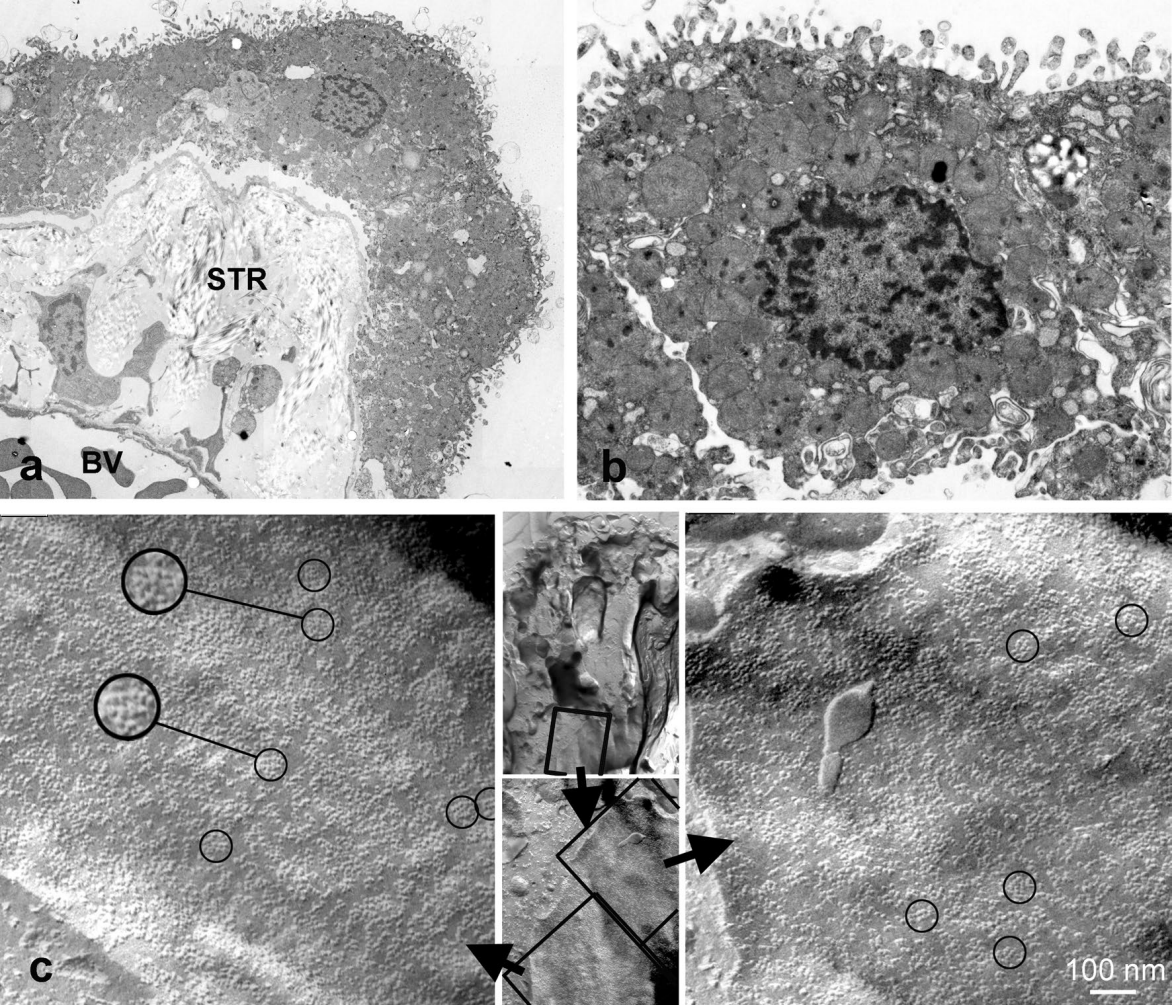
Electron micrographs of human CP epithelial cells in ultrathin sections (**a**, **b**) and freeze fracture preparations (**c**). Note the extensive stroma (STR) between blood vessels (BV) and epithelial cells. In **c**, the high power views revealed orthogonal arrays of particles (circles) which are known to be formed by AQP4. The inserts in the middle indicate the location of the membrane faces on the CP epithelial cell

We performed ultrastructural analysis since AQP4 is known to form orthogonal arrays of particles (OAPs) in freeze-fracture electron microscopy. The analysis of ultrathin sections revealed an abundance of collagen fibers in the stroma underneath the CP epithelial cells. Most of CP epithelial cells displayed microvilli, basal membrane foldings, mitochondria, and many vesicular bodies (Fig. 3a, b). In freeze-fracture replicas, we detected OAPs in membranes of CP epithelial cells (Fig. 3). This is additional evidence for the presence of AQP4 in the human CP.

### Co-localization of aquaporins with NKCC1 and Na/K-ATPase

**Fig. 4:**
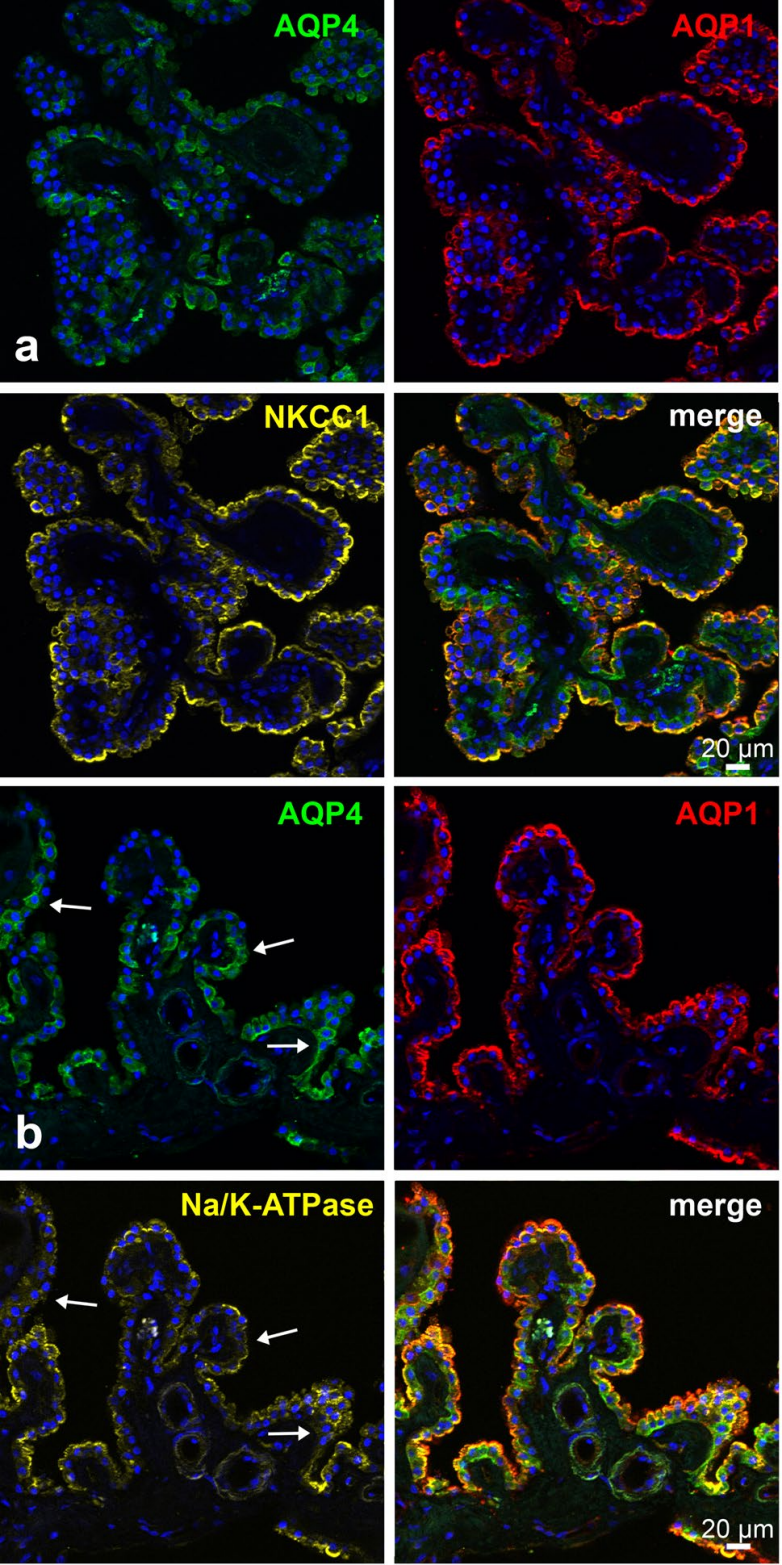
Triple immunostains for transport proteins and aquaporins in the human CP. Cell nuclei were counterstained with Draq5 (blue). **a** Immunostains for AQP4, AQP1, and the Na^+^-K^+^-2Cl^−^ cotransporter NKCC1. NKCC1 showed an almost continuous homogeneous expression on the apical side of the plexus epithelium, which was colocalized with AQP1. Vice versa, most AQP4-positive cells also showed expression of NKCC1. **b** Immunostains for the Na^+^/K^+^-ATPase, AQP1, and AQP4. Na^+^/K^+^-ATPase was expressed apically yet not as homogeneously as the apical AQP1 expression, nor the apical NKCC1 expression shown in a. In areas (arrows) where AQP4-positive cells were present, there was weaker or lacking Na/K-ATPase immunoreactivity. AQP1 showed continuous expression

To find out how this unusual AQP4 expression relates to the expression of relevant transport proteins we expanded our analysis with stainings for NKCC1 (Fig. 4a) and Na/K-ATPase (Fig. 4b) in our analysis. NKCC1 showed an almost continuous homogeneous expression on the apical side of the plexus epithelium, which was colocalized with AQP1. Vice versa, most AQP4-positive cells also showed expression of NKCC1. Like NKCC1, Na/K-ATPase was expressed apically but not homogeneously: There was a tendency that Na/K-ATPase and AQP4 were inversely distributed, more specifically, there were areas (arrows in Fig. 4b) where AQP4- positive cells did not reveal Na/K-ATPase immunoreactivity. AQP1 showed continuous apical expression. This result could be confirmed in the CP tissue of all body donors investigated.

### Changes of AQP4 expression in aging

**Fig. 5:**
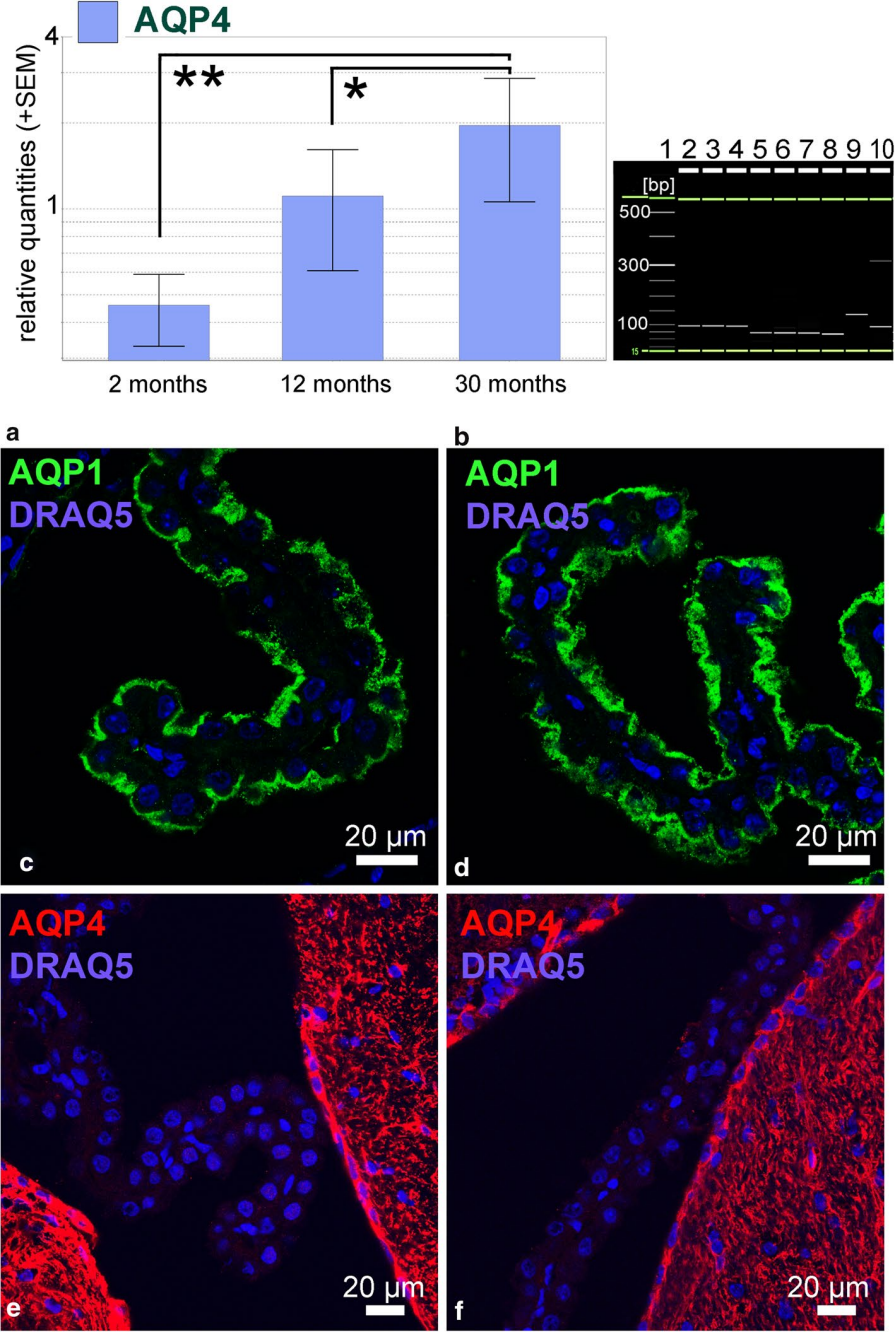
AQP4 expression in the mouse choroid plexus from different age groups. **a.** Quantitative RT-PCR analysis using TaqMan^®^ gene expression assays for mice of three different ages (n = 3 for each group), HPRT, TBP and UBC served as reference genes and the striatum as reference tissue. The relative mRNA expression of AQP4 based on the qbase + exported relative quantity (RQ) values, calculated from Cq values. Qbase + results are scaled to the average across all unknown samples per target showing the relative quantity for AQP4 was significantly higher in the 30 month-old group compare to the younger groups (* indicates p < 0.05, ** p < 0.0005). **b** Exemplary gel image of RT-PCR products obtained from QIAxel system software showing the expected product size for AQP1 (94 bp), AQP4 (69 bp), HPRT (65 bp), TBP (138 bp), UBC (92 bp). Lane 1-size marker, 2-AQP1 2-months, 3-AQP1 12-months, 4-AQP1 30-months, 5-AQP4 2-months, 6-AQP4 12-months, 7-AQP4 30-months, 8-HPRT 2-months, 9-TBP 2-months, 10-UBC 2-months. **c–f** Immunofluorescence staining for AQP1 (**c, d**) and AQP4 (**e, f**) in CP tissue from the lateral ventricle of young, 2 months old mice (**c, e**), and old, 30 months old mice (**d, f**). AQP1 was apically expressed in CPCs with no obvious difference between age groups. AQP4 immunofluorescence was not detected on CPCs in neither young nor older mice, with strong reactivity in adjacent ependymal and subependymal regions

Since all our body donors were more than 70 years old, we hypothesized that AQP4 expression in the CP was caused by age-related changes. To address this, we used TaqMan^®^ Gene Expression assays for quantitative real-time PCR analysis in mice of various ages. We investigated mouse brains from young (2 months), adult (12 months), and aged (30 months) mice. We analyzed five biological replicates for each age cohort and found that the relative amount of AQP4 mRNA in older mice was significantly increased (Fig. 5a). We confirmed the specificity of amplified PCR products by QIAxcel high-resolution capillary electrophoresis showing the expected product size for AQP1, AQP4, NKCC1, and the three reference genes (Fig. 5b). We also stained tissue from the three age groups for AQP1 and AQP4. AQP1 was located apically in CPCs with no apparent differences between age groups (Fig. 5c, d). We did not find AQP4 positive cells in the CP of old mice. Thus, although AQP4-RNA was present and increased in older mice, the AQP4 protein does not seem to be expressed at levels detectable by immunofluorescence (Fig. 5e, f).

## Discussion

In this study we report the expression of the water channel AQP4 in the human choroid plexus. In each of the 8 body donors, AQP4-positive cells could be visualized by immunofluorescence in the epithelium of the CP. In addition, we found OAPs formed by AQP4 on the ultrastructural level. This was a surprising result since plexus epithelial cells so far have been known to express AQP1 only [2, 12, 25], whereas ventricle-lining ependymal express only AQP4 [26, 27]. Although there have been reports on the presence of AQP4 in the CP of rats, these studies showed either a weak in-situ hybridization signal [28], or a diffuse cytoplasmic immunofluorescence reactivity [29] in the choroid plexus. Our results, however, show a mostly basolateral expression of AQP4 in many CPCs. Furthermore, we showed the expression of AQP4 at the mRNA level for the human choroid plexus. The post-mortem interval for the three body donors used for quantitative RT-PCR of 11 h or less is well within the range when suitable RNA is still preserved in the brain [30, 31] and was confirmed by the RIS values.

As a possible cause for the appearance of AQP4 in the plexus epithelium we suspected a relationship with the process of aging. Indeed, we did observe presumably age-related characteristics such as psammoma bodies [32]. In addition and consistent with previous studies [33, 34], we found extensive connective tissue in the thickened CP stroma which very likely results in an impaired diffusion since the distance between plexus epithelium and blood vessels is increased (see Figs. 1 and 3). Lower expression of AQP1 has been reported in aging rats [35], and a decrease in CSF production was observed in old sheep (7–10 years) compared to young sheep (1–2 years), with an increase in protein CSF/plasma ratio while protein plasma levels remained constant [36]. In addition, a decreased CSF production has been suggested to play a role in Alzheimer’s disease [34, 37], and a lower CSF flow in elderly patients has been linked to cognitive impairment [38]. Thus, age-related changes in the CP seem to be more pronounced in Alzheimer’s disease (for a recent review see [21, 39].

In the context of these morphological and functional changes associated with age and disease in the CP, the detection of AQP4 in CPCs and can be interpreted in two alternative scenarios: First, AQP4 expression could serve as a compensatory mechanism in old age to maintain CSF production known to be decreased. The compensation could be implemented by constitutive expression of AQP4 or a regulated mechanism similar to that described for antidiuretic hormone (ADH) and aquaporin-2 in the kidney. Here, antidiuretic hormone (vasopressin) which is produced in the hypothalamus, activates a signaling cascade resulting in AQP2 membrane incorporation [40]. A similar process could regulate AQP4 in the CP at low CSF production levels since there was immunoreactivity for AQP4 in the cytoplasm of some cells. Although such a mechanism has not been verified for AQP4 so far, vasopressin has been reported to modulate water flux in the cerebral cortex likely via AQP4 [41]. Recently, it was shown that AQP4 expression in the mouse CP can be induced by experimental hypoxic conditions [42]. Moreover, cytoplasmic isoforms of human AQP4 generated by alternative splicing have been shown to influence AQP4 membrane expression [43] and might affect the formation of OAPs and therefore water homeostasis [44].

**Fig. 6:**
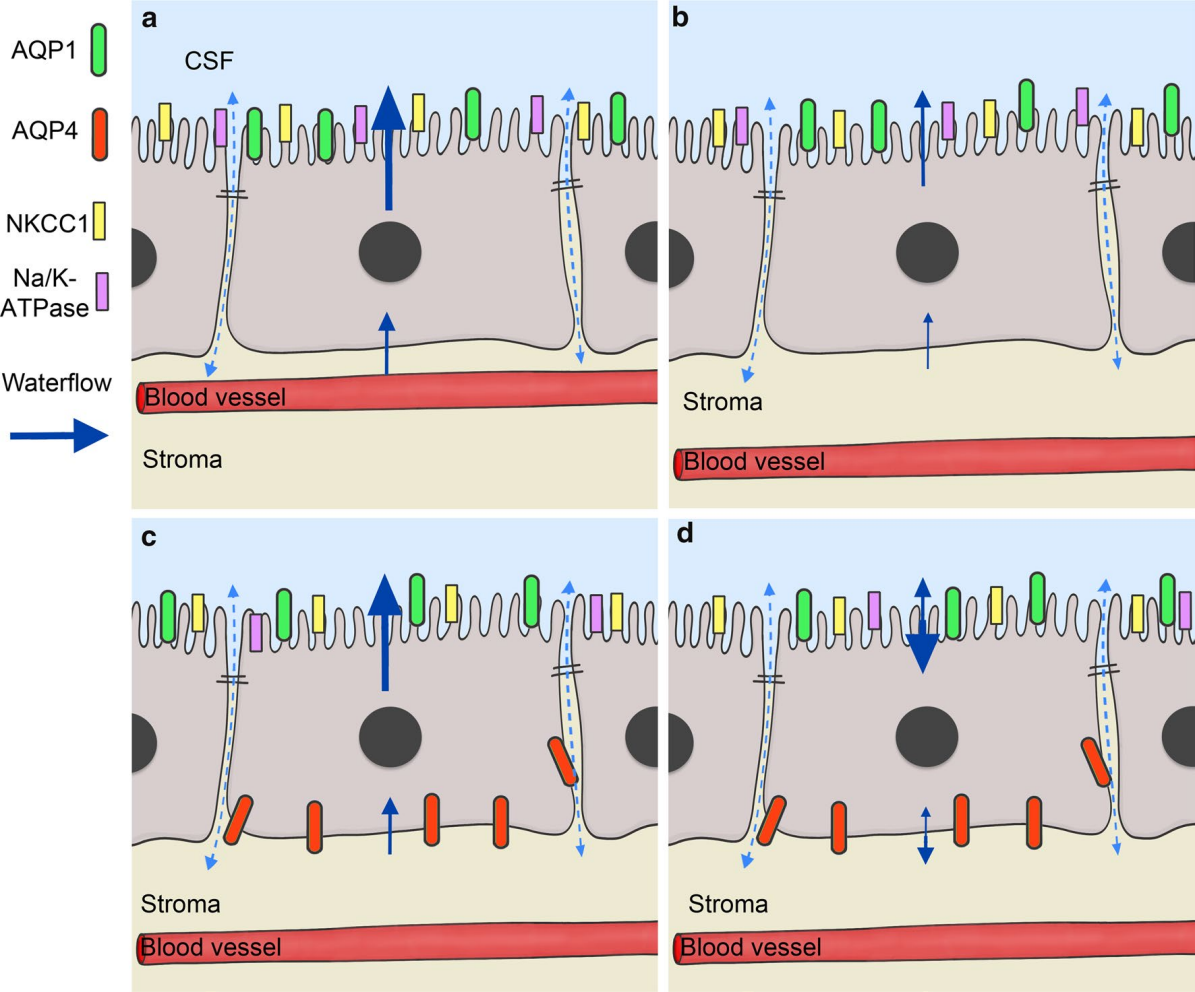
A graphical illustration of the channels and transporters examined in this study, and their suggested impact on the waterflow and CSF production in aging. **a** A choroid plexus epithelial cell in an adult human with normal waterflow and CSF production. **b ** An aged choroid plexus epithelium cell with reduced waterflow and reduced CSF production without basolateral AQP4 expression. Note increased diffusion distance between the blood vessel and the epithelium. **c **Explains our first hypothesis: AQP4 is expressed to compensate the reduced CSF production in aged humans. The CP cells express AQP4 basolaterally to generate a higher transcellular waterflow. This leads to a normal waterflow and CSF production. **d** Explains our alternative hypothesis: The expression of AQP4 leads to an inverted transcellular waterflow carrying a reduced CSF production with it

An alternative hypothesis can be inferred from AQP4 expression in the ependyma adjacent to the plexus epithelium. Here, AQP4 is expressed in the basolateral membrane domain of ependymal cells [11, 14]. The basolateral expression corresponds to the histological localization we found in CPCs. Therefore, CPCs might differentiate over time into AQP4-positive cells taking on characteristics of ependymal cells. This could include a partial basolateral instead of apical water outflow along an osmotic gradient [45] and would be consistent with reduced CSF production in the aging brain. The two hypotheses about the possible consequences of AQP4 expression in the aging CP affecting water flow are illustrated in Fig. 6.

Since CP tissue of young and healthy human adults is difficult to obtain, we turned to a mouse model to compare different age groups. Indeed, we could observe an increase in the AQP4/AQP1 ratio in correlation to the age but only at the mRNA level. In contrast, antibody staining for AQP4 was negative in the CP epithelium of old mice. Thus, it is possible that the translation of AQP4 protein is suppressed by some regulatory mechanisms in the mouse. For example, several miRNAs have been reported to downregulate the expression of AQP4 (summarized in [46]). More recently, the RNA binding protein DDX4 has been identified as a negative regulator of AQP4 translation in mice [47]. Consistent with our results, Trillo-Contreras et al. [42] reported expression of AQP4 in the CP especially of aged mice under hypoxic condition on the mRNA level, and even moderately on the protein level.

The reasons for the difference in human and mouse AQP expression on the protein level observed in this study could be due to a variety of reasons. Obviously, the maximum life span of the mouse (3–4 years) differs greatly with that of humans (≥ 100 years [48]), yet a recent MRI study showed a clear reduction of water delivery through the CP to the ventricles in aged mice [49]. However, the macroscopic and microscopic structure of the CP of the mouse differs from that of the human. While the CP of the lateral ventricle in mice consists of a thin vascular layer covered by CP epithelium on both sides [50], the human CP is a highly branched structure with villi and extensive connective tissue. In addition, we observed thickened subepithelial stroma, and deposits such as psammoma bodies (see Fig. 1b) not found in the CP of old mice. Thus, changes occurring with age might be compensated for in other ways in the mouse, and despite increased mRNA levels, the AQP4 channel is not incorporated into the plexus epithelium. In humans, our data show not only the occurrence of AQP4 at the mRNA level but also at the protein level as basolateral expression in the CP epithelium.

Either one of the proposed scenarios shown in Fig. 6 would also have an impact on the so-called 'glymphatic pathways', which describes an exchange of cerebrospinal fluid and interstitial fluid as well as metabolites in the brain [51]. The transport is thought to take place peri-arterially and peri-venously, and then via the AQP4 endfeet of astrocytes. CSF is taken up by the astrocytes via AQP4, and dissolved substances are washed out paracellularly [52]. Such CSF uptake from the ventricles and delivery to the CP stroma could be enabled by AQP4. An MRI study with aquaporin-KO mice suggested a higher contribution to CSF production by AQP4 than AQP1 [53].

The direction of water flow through aquaporins is determined by the osmotic and hydrostatic gradient [54]. By decreasing Na/K-ATPase [35] (this study Fig. 5) thus lowering oncotic pressure, CSF could be absorbed from the ventricles in the reversed direction. In a recent study [45], explants from rat CP provide evidence for a substantial amount of apical outward water flow through the NKCC1 co-transporter, in addition to AQP1. Another report on isolated CP cells, however, suggest an inward flow through NKCC1 to contribute to cell water volume maintenance needed for CSF secretion [55]. This debate is far from settled (see recent discussions [56, 57]). Our data of the expression of the fast water channel AQP4 in the CP suggests that the water balance across the epithelium can be changed, and it remains to be shown whether this is a beneficial compensatory mechanism or not. To confirm one of these alternatives, more investigations on isolated CP tissue or cells, and further age comparisons of CP tissues will be necessary.

## Conclusions

In summary, we could demonstrate the expression of AQP4 in the epithelium of the human CP and age-related changes in the murine CP on the RNA level. The expression of AQP4 in the aging CP has consequences for the models of water flow through the CP epithelium discussed so far. Thus, an increased water inflow though AQP4 could compensate for impaired perivascular diffusion; alternatively, AQP4 in the CP could be misexpressed and slow down CSF production.

## Supplementary Information

Below is the link to the electronic supplementary material.

Supplementary file1 (DOCX 16 KB)

Supplementary file2 (DOCX 15 KB)

Supplementary Figure 1 Gene expression analysis for AQP1, AQP4, and NKCC1 using TaqMan® assays of the striatum from three human body donors, HPRT, TBP and UBC served as reference genes. AQP4 mRNA levels were high in all body donors (compare Fig. 2b) whereas mRNA levels for AQP1 were very low (PNG 54 KB)

## Data Availability

The datasets used and/or analysed during the current study are available from the corresponding author on reasonable request.

## Abbreviations

AQP1: Aquaporin-1
AQP4: Aquaporin-4
BCSFB: Blood-cerebrospinal fluid barrier
CNS: Central nervous system
CP: Choroid plexus
CPCs: Choroid plexus epithelial cells
CSF: Cerebrospinal fluid
NKCC1: Na+–K+–2Cl−cotransporter1
OAPs: Orthogonal arrays of particles
