# Supplementary material for: Aquaporin-4 expression in the human choroid plexus
Source: Cell Mol Life Sci. 2022 Jan 24;79(2):90. doi: 10.1007/s00018-022-04136-1 (PMC8785037; doi:10.1007/s00018-022-04136-1)
Supplement: Supplementary file 1 — Supplementary file1 (DOCX 16 KB) [file 18_2022_4136_MOESM1_ESM.docx]

| **Gen-alias** | **species** | **Assay number** | **Amplicon length (bp)** |
| --- | --- | --- | --- |
| HPRT | mouse | Mm00446968_m1 | 65 |
| TBP | mouse | Mm01277045_m1 | 138 |
| UBC | mouse | Mm01201237_m1 | 92 |
| AQP1 | mouse | Mm01326466_m1 | 94 |
| AQP4 | mouse | Mm00802131_m1 | 69 |
| HPRT | human | Hs02800695_m1 | 82 |
| NKCC1 | human | Hs00169032_m1 | 97 |
| TBP | human | Hs00427620_m1 | 91 |
| UBC | human | Hs00824723_m1 | 71 |
| AQP1 | human | Hs01028916_m1 | 96 |
| AQP4 | human | Hs00242342_m1 | 92 |

**SM table 2:** TaqMan® Assays
