# Supplementary material for: Aquaporin-4 expression in the human choroid plexus
Source: Cell Mol Life Sci. 2022 Jan 24;79(2):90. doi: 10.1007/s00018-022-04136-1 (PMC8785037; doi:10.1007/s00018-022-04136-1)
Supplement: Supplementary file 2 — Supplementary file2 (DOCX 15 KB) [file 18_2022_4136_MOESM2_ESM.docx]

| **age** | **sex** | **post mortem interval** | **cause of death*** |
| --- | --- | --- | --- |
| 85 years | male | 11 h | kidney failure |
| 83 years | female | 15 h | pulmonary carcinoma |
| 89 years | male | 12 h | kidney failure |
| 91 years | female | 19 h | exsiccosis, GI bleeding |
| 93 years | male | 11 h**^#^** | pneumonia |
| 75 years | female | 8 h**^#^** | ischemic stroke |
| 74 years | female | 9 h**^#^** | multi organ failure |
| 94 years | female | 11 h | cardiac arrest |

**SM Table 1:** Age, sex and postmortem interval of body donors used in this study.

* cause of death as stated in the official death certificate after external examination

**^#^** indicates the body donors used for RNA isolation
