# Supplementary figures and images for: Aquaporin-4 expression in the human choroid plexus
Source: Cell Mol Life Sci. 2022 Jan 24;79(2):90. doi: 10.1007/s00018-022-04136-1 (PMC8785037; doi:10.1007/s00018-022-04136-1)

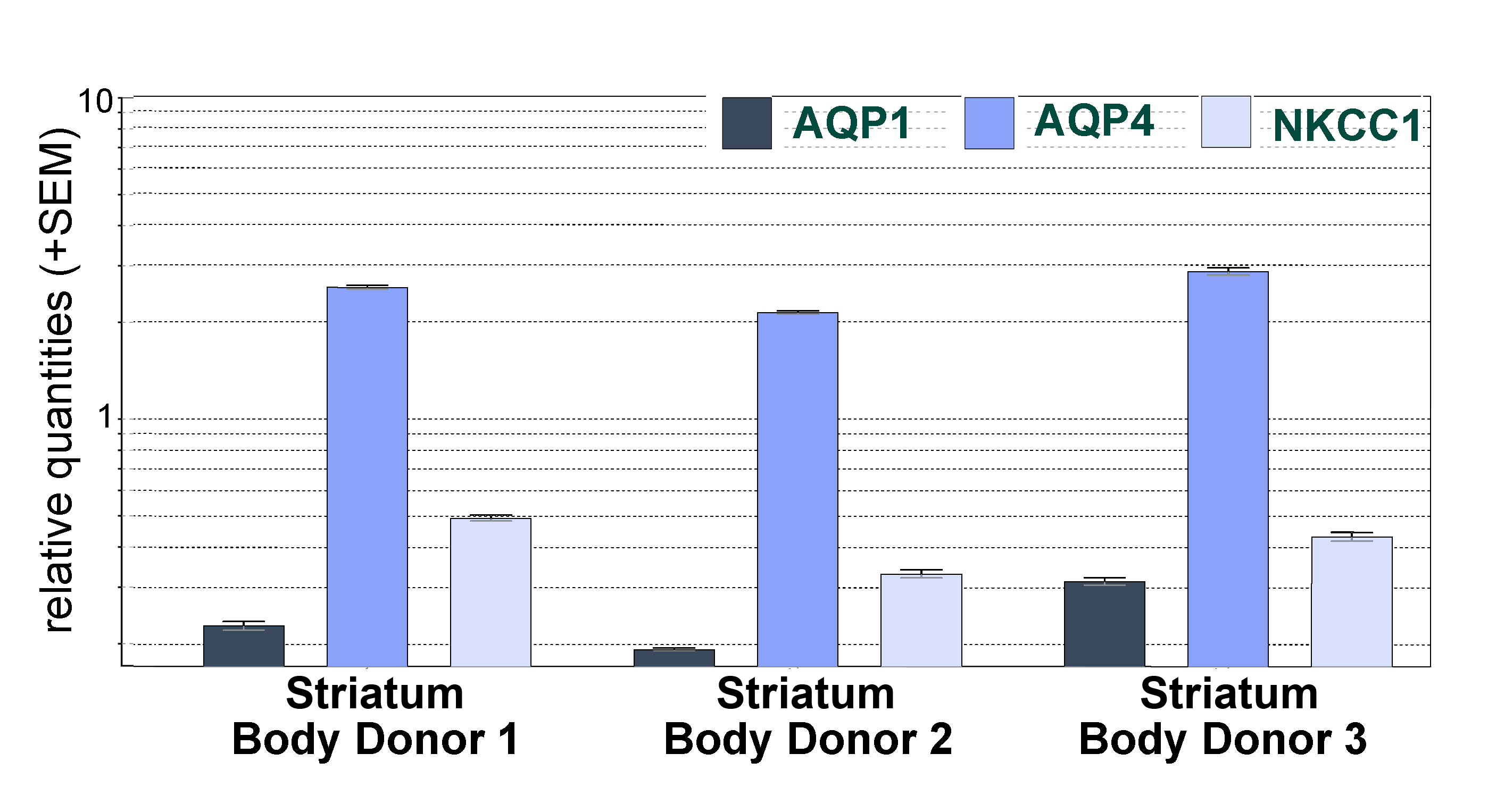

Supplement: Supplementary file 3 — Supplementary Figure 1 Gene expression analysis for AQP1, AQP4, and NKCC1 using TaqMan® assays of the striatum from three human body donors, HPRT, TBP and UBC served as reference genes. AQP4 mRNA levels were high in all body donors (compare Fig. 2b) whereas mRNA levels for AQP1 were very low (PNG 54 KB) [file 18_2022_4136_MOESM3_ESM.png]
